# Tomographic Imaging and Localization of Nanoparticles in Tissue Using Surface-Enhanced Spatially Offset Raman Spectroscopy

**DOI:** 10.1021/acsami.2c05611

**Published:** 2022-07-08

**Authors:** Matthew E. Berry, Samantha M. McCabe, Sian Sloan-Dennison, Stacey Laing, Neil C. Shand, Duncan Graham, Karen Faulds

**Affiliations:** † Department of Pure and Applied Chemistry, Technology and Innovation Centre, University of Strathclyde, 99 George Street, Glasgow G1 1RD, U.K.; ‡ The Defence Science and Technology Laboratory (Dstl), Porton Down, Salisbury SP4 0JQ, U.K.

**Keywords:** Raman, SERS, SORS, SESORS, nanoparticles, imaging, tissue

## Abstract

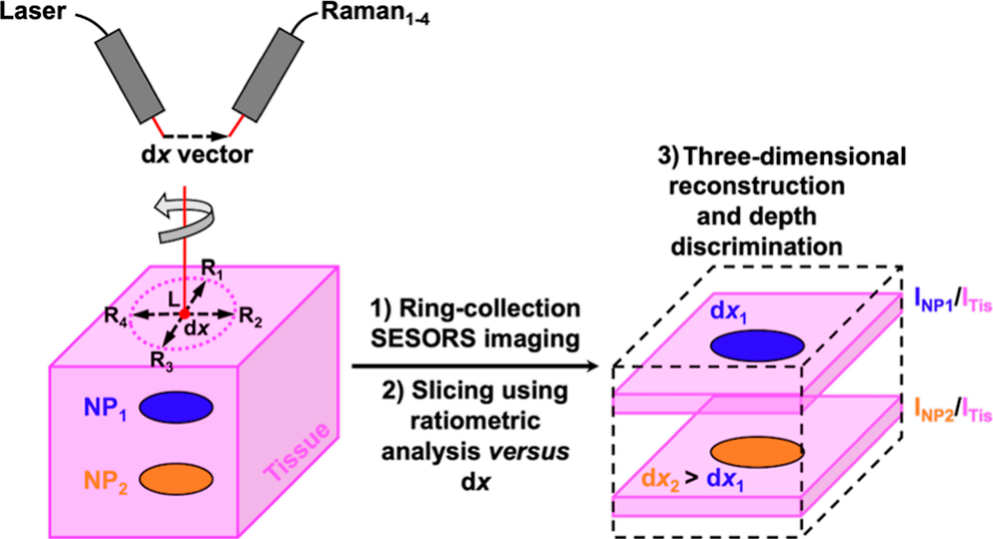

A fundamental question crucial to surface-enhanced spatially offset Raman spectroscopy (SESORS) imaging and implementing it in a clinical setting for in vivo diagnostic purposes is whether a SESORS image can be used to determine the exact location of an object within tissue? To address this question, multiple experimental factors pertaining to the optical setup in imaging experiments using an in-house-built point-collection-based spatially offset Raman spectroscopy (SORS) system were investigated to determine those critical to the three-dimensional (3D) positioning capability of SESORS. Here, we report the effects of the spatial offset magnitude and geometry on locating nanoparticles (NPs) mixed with silica powder as an imaging target through tissue and outline experimental techniques to allow for the correct interpretation of SESORS images to ascertain the correct location of NPs in the two-dimensional *x*, *y*-imaging plane at depth. More specifically, the effect of “linear offset-induced image drag” is presented, which refers to a spatial distortion in SESORS images caused by the magnitude and direction of the linear offset and highlight the need for an annular SORS collection geometry during imaging to neutralize these asymmetric effects. Additionally, building on these principles, the concept of “ratiometric SESORS imaging” is introduced for the location of buried inclusions in three dimensions. Together these principles are vital in developing a methodology for the location of surface-enhanced Raman scattering-active inclusions in three dimensions. This approach utilizes the relationship between the magnitude of the spatial offset, the probed depth, and ratiometric analysis of the NP and tissue Raman intensities to ultimately image and spatially discriminate between two distinct NP flavors buried at different depths within a 3D model for the first time. This research demonstrates how to accurately identify multiple objects at depth in tissue and their location using SESORS which addresses a key capability in moving SESORS closer to use in biomedical applications.

## Introduction

Surface-enhanced spatially offset Raman spectroscopy (SESORS) is an emerging analytical technique that combines the Raman signal enhancements afforded by surface-enhanced Raman scattering (SERS) with the through-barrier detection capabilities of spatially offset Raman spectroscopy (SORS).^1−8^ The combination of these techniques to produce SESORS enhances the potential of translating Raman-based techniques to in vivo detection of various disease states in a clinical setting.^9−13^ SORS spectrometers make use of an applied spatial offset, the magnitude of which is denoted as d*x*, on the surface of an obscuring barrier between excitation and collection points, removing the surface selectivity of measurements conducted using traditional, single-probe Raman systems.^14^ The application of a spatial offset on the surface of the obscuring barrier during measurements allows for the collection of Raman photons that have been scattered by subsurface analytes, and the delineation of compositional changes at depth is possible when comparing spectra collected at offsets of different magnitudes.^15^ There are various approaches that allow excitation of multilayered samples, such as mammalian tissue analogues containing buried SERS-active nanoparticles (NPs), and the offset collection of subsurface-scattered Raman photons, as highlighted in a recent review article by Nicolson et al.^16^ These techniques span numerous different optical geometries including point-based collection SORS, where the point of collection is offset from the point of excitation by a linear offset vector; ring-collection SORS, where the Raman photons are collected in a circular array around a central excitation point and the spatial offset magnitude is calculated as the radius of the collection ring; and transmission Raman spectroscopy, where the excitation and collection points are situated on the opposite sides of a sample.^17^

In recent years, SESORS has been explored as an imaging technique for the detection of various disease states through biological tissues via the interaction of biomolecular targets with biofunctionalized SERS-active NPs that produce highly specific molecular fingerprint spectra. Imaging is traditionally undertaken by tracking the intensity of a particular SERS band, pertaining to the targeted NPs of interest, across a grid on the surface of a biological sample to ascertain the location of the NPs at depth in the two-dimensional (2D) imaging plane. Using a handheld SORS spectrometer with a point-based collection geometry, that is, a linear offset vector, Nicolson et al. reported the through tissue imaging of ex vivo MCF7 multicellular tumor spheroid (MTS) models incubated with SERS-active gold NPs. In this study, the MTS models were buried beneath the porcine tissue and mapped in a grid across the tissue surface using a handheld spectrometer.^18^ Furthermore, the multiplexed detection and imaging of multiple SERS-active gold NPs incubated within the MTS models through porcine tissue was reported using principal component analysis to discriminate between different Raman reporters.^19^ SESORS imaging of brain cancer has also crucially been translated to in vivo use, with the noninvasive imaging of glioblastoma multiforme (GBM) being demonstrated in living mice using silica-capped gold nanostars functionalized with Raman reporter molecules and coated on the outside with GBM-targeting cyclic RGD peptides.^20^ This work also utilized a SORS geometry with a linear offset vector, but in this instance, the spectrometer consisted of two probes on a fixed benchtop system.

For SESORS imaging to be considered as a viable optical medical imaging method, it is crucial to not only apply the technique to different assays and disease states but also to cultivate theoretical principles for the correct interpretation of such images and, hence, the accurate location of targeted NPs in vivo at depth in three dimensions. We sought to address a fundamental question relevant to all the SESORS imaging studies alluded to here, namely, how does a SESORS image, collected at points offset from the excitation points on an imaging grid by a constant spatial offset, relate to the physical location of a SERS-active inclusion through tissue? We have conducted a study that investigates multiple factors pertaining to the optical settings within imaging experiments on an in-house-built SORS system to address this question.^21^

Herein, we report the effects of the magnitude of the spatial offset and the nature of the geometry in locating SERS-active NPs in a novel SESORS imaging template through tissue and outline experimental techniques to allow for the correct interpretation of SESORS images and the location of NPs in the 2D *x*, *y*-imaging plane at depth. More specifically, we present the effect of “linear offset-induced image drag” which refers to a spatial distortion in SESORS images caused by the magnitude and direction of the linear offset vector and highlight the need for a radial SORS collection geometry during imaging to neutralize effects such as asymmetric image artifacts that would otherwise lead to misinterpretation of data and incorrect identification of the inclusion location. Additionally, building on these principles, we simultaneously introduce the concept of “ratiometric SESORS imaging” for the location of buried inclusions in three dimensions. This technique differs from the more traditional form of SESORS imaging, in which the intensity of a specific SERS band from the subsurface NPs is used to quantify each pixel, in that the relative contribution of the NP SERS band to the through tissue spectrum, *I*_NP_/*I*_Tis_, is used to quantify the value of each pixel to build the image. This ratiometric technique was first reported by Asiala et al. in a study that utilized spin-coated SERS-active NP samples to monitor *I*_NP_/*I*_Tis_ as a function of tissue barrier thickness and magnitude of a linear offset vector and indicated a positive relationship between the thickness of the tissue barrier and the offset magnitude required to access inclusions behind those barriers.^21^ Mosca et al. reached a similar conclusion using Monte Carlo simulations and stipulated that when analyzing a turbid sample with a point-based collection SORS geometry, it is possible to relate the magnitude of the linear offset vector to the depths that are being probed by the laser.^22^ These principles are vital in developing a methodology for the location of SERS-active inclusions in three dimensions, and we test the robustness of our approach, which utilizes the relationship between the magnitude of the spatial offset, the probed depth, and the ratiometric analysis, *I*_NP_/*I*_Tis_, to ultimately image and spatially discriminate between two distinct NP flavors buried at different depths in three dimensions for the first time.

## Methods

### Materials and Equipment

Sodium tetrachloroaurate(III) dihydrate, trisodium citrate, 4-(1*H*-pyrazol-4-yl)pyridine (PPY), 1,2-bis(4-pyridyl)ethylene (BPE), methanol, and (3-aminopropyl)triethoxysilane (APTES) were purchased from Sigma-Aldrich. Milli-Q water (MQ H_2_O) was prepared in-house. Sodium silicate in aqueous solution and a silica gel (40–63 μm) were purchased from VWR chemicals. The Raman reporters, BPE and PPY, were prepared in stock solutions by dissolving the solid in methanol. Subsequent dilutions were then carried out in MQ H_2_O. Lean pork back tissue was used as a mammalian tissue analogue. Samples were purchased from a local supermarket and cut into sections (roughly 50 mm × 50 mm with a 3 mm thickness).

**Figure 1:**
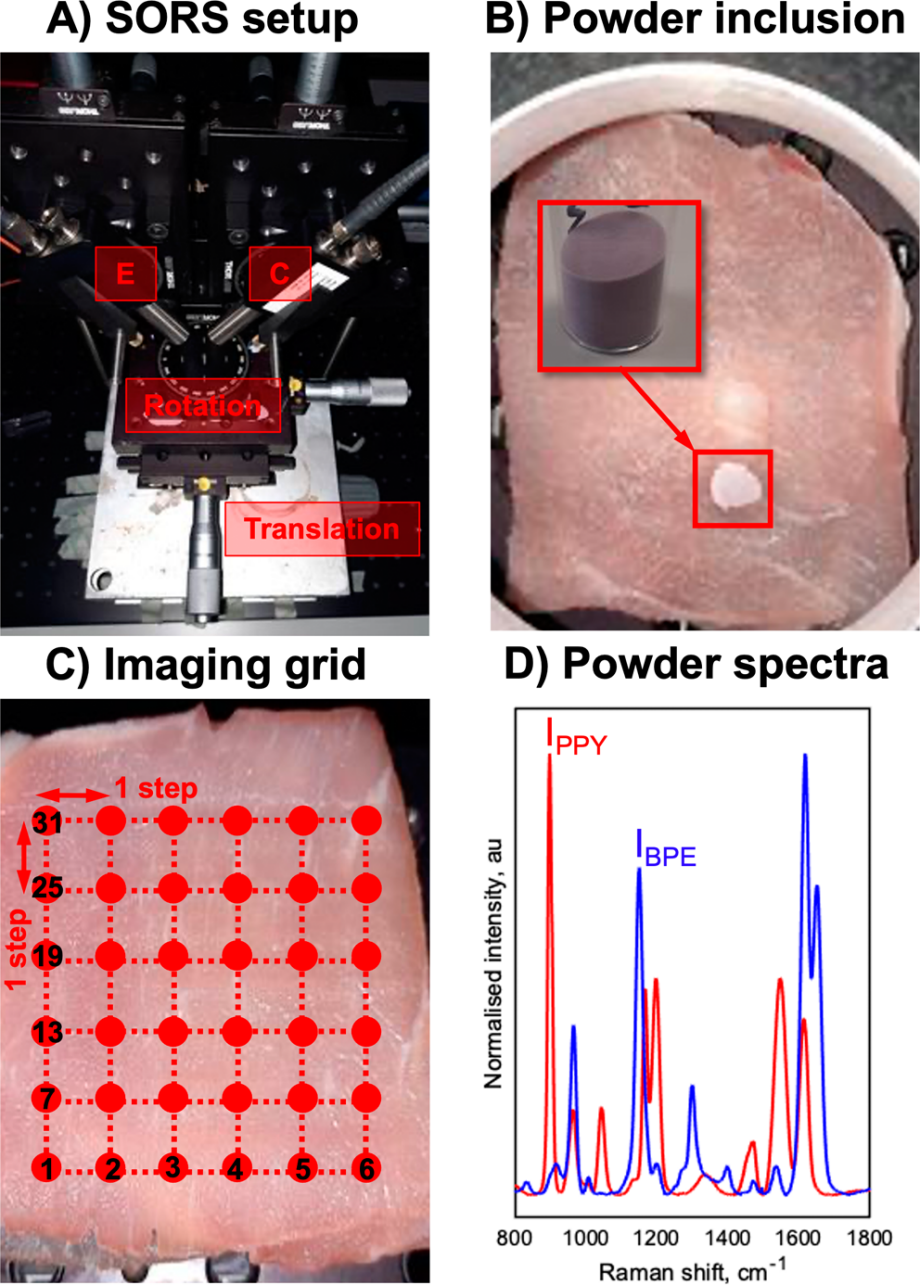
(A) SESORS experiments were conducted on an in-house-built SORS system with a point-based collection geometry, i.e., with individual excitation (E) and collection (C) probes, developed by Asiala et al. based on the design of Shand and co-workers.^21,23^ (B) Templates for SESORS experiments were prepared by mixing the silica powder with silica-coated SERS-active NPs. The NP powders were then added to a section of the porcine tissue and then covered with further tissue sections. (C) Hypothetical SESORS imaging regime illustrated using a square grid, where each vertex on the grid represents a pixel where the tissue surface is excited by the laser. In the regime illustrated, the grid represents a 36-pixel (6 × 6) image. (D) Truncated, baselined, and normalized SERS spectra of PPY (red) and BPE (blue) powder-based imaging templates developed for this study. Spectra were collected for 0.1 s using a 785 nm, 400 mW laser.

A Cary 60 UV–Visible spectrophotometer with a wavelength range of 300–800 nm and a scan rate of 24,000 nm/min was used to acquire extinction spectra, which were used to characterize the optical properties of the NPs. A Malvern Zetasizer nano ZS system was used to conduct dynamic light scattering (DLS) and zeta potential measurements. Samples were added to a poly(methyl methacrylate) cuvette with 1 cm path length for all measurements. For zeta potential measurements, a dip cell was placed in the cuvette. SESORS experiments were conducted on an in-house-built SORS system with a point-based collection geometry, developed by Asiala et al. based on the design of Shand and co-workers and is shown in Figure 1A.^21,23^ A 785 nm laser (Innovative Photonics Solutions) with an attenuable output was coupled to one of the two fiber optic Raman probes (Wasatch Photonics Ltd) with built-in filtering optics. The probes were mounted on independent *x*, *y*, *z*-translational stages with rotational mounts (ThorLabs) for accurate positioning and as a convenient means of directly measuring the spatial offset magnitude. The collection probe was coupled to a *f*/1.3 Raman spectrometer (WP 785, Wasatch Photonics Ltd), and the excitation and collection probes were angled at approximately 60° from each other. Spectra were collected for 1 s using a laser power of 400 mW. It is worth commenting that the maximum permitted exposure for the skin is exceeded with the laser settings used and that the settings were selected primarily to improve the through tissue signal of the NPs for demonstration purposes. Since the intended application of this work is in vivo biomedical sensing, any future clinical studies would have to account for this factor.

### Sample Preparation

Gold NP cores with an average size between 50 and 60 nm were synthesized using a modified Turkevich method where citrate ions act as the reducing and capping agents for the NPs.^24^ Sodium tetrachloroaurate(III) dihydrate (70 mg) was added to MQ H_2_O (500 mL) and heated to boiling. Trisodium citrate (42 mg) was added to MQ H_2_O (7.5 mL), and the two solutions were combined. The resulting solution was boiled for 15 min with continuous stirring and then allowed to cool. SERS-active silica-coated NP aggregates were synthesized using a method modified from the study by Li et al.^25^ Raman reporters (300 μL, 100 μM) were added separately to solutions of AuNPs (100 mL) under stirring. The solution was stirred until aggregation occurred, and the SERS response was optimized. APTES (1.6 μL, undiluted) was then added, followed immediately by sodium silicate (111 μL, undiluted). The mixture was stirred vigorously at 90 °C for 30 min, and it was then removed from heat and stirred overnight. The NPs were then centrifuged (7300*g*, 20 min) and resuspended in MQ H_2_O (30 mL). Powder-based SERS-active imaging templates, shown in Figure 1B, were then prepared by mixing an aliquot of silica-coated aggregates (9 mL) with a silica powder (2 g). The mixture was placed in a 60 °C oven and dried for 1 h, and the resulting powder was placed in a sealed container for future use.

### SESORS Imaging

SESORS imaging was performed on an in-house-built SORS system with a point-based collection geometry. Approximately 30 mg of the powder-based SERS-active NP imaging templates was transferred and placed on top of a section of a porcine tissue, Figure 1B. The powder was then covered by another section of the porcine tissue; multiple depths were used, and each of the following sections stipulates the specific depth used for each experiment to ensure that the powder was obscured from the optical setup. A translational *x*–*y* stage with a range of 2.54 cm was used to maneuver the tissue samples in the *x*, *y*-imaging plane, Figure 1A,C. Images were collected throughout the study by recording pixels across rows in a particular direction and then moving the samples incrementally through each row in the orthogonal axis. The step sizes and pixel numbers were varied across the study, and in each of the following sections, both parameters are specified with regard to each experiment. The samples were brought into focus with the excitation and collection probes using a *z*-translational stage below the imaging stage. The samples were focused by raising the stage vertically with the laser and spectrometer operational until the tissue contribution to the resulting SORS spectrum was maximized, that is, by tracking the intensity of the 1425 cm^–1^ CH_2_ scissoring band characteristic of lipids in mammalian tissues. This indicated that the excitation and collection probes were focused on the same spot on the tissue surface because the relative contribution of the surface was maximized in the resulting spectra. To mimic a ring-collection offset, a rotational mount, shown in Figure 1A, was placed on top of the combined *x*, *y*, *z*-translational stage to allow for rotation of the samples four times through 360° in 90° steps and simulation of a rotating linear offset vector. Images were recorded between each rotation in the manner shown in Figure 1C, and the resulting four images were then averaged at each pixel, generating an image with a simulated ring-collection offset.

### Data Processing

All spectra were processed using MATLAB software (Version 2018b, The MathWorks, Natick, MA, USA). Preprocessing involved truncating and baselining the spectra which corresponded to image pixels. For intensity images, the SERS intensity of the NPs (905 and 1154 cm^–1^ for PPY and BPE, respectively, based on Raman shift calibrations) was plotted at each pixel as a false-color 2D heat map. For ratiometric images, the SERS intensity of the NPs was divided by the maximum Raman intensity of the tissue (1425 cm^–1^) across all pixels in the image (*I*_NP_/*I*_Tis max_). These values were then plotted at each pixel as false-color 2D heat maps. Where stated, the color bars across each image in a set of ratiometric images were scaled for clarity, for example, in a fixed depth and variable offset experiment to allow for three-dimensional (3D) sectioning of the tissue block. To mimic a ring-collection offset, ratiometric images were collected as the sample was rotated through 360° in 90° steps to simulate rotation of the linear offset vector. Since the samples were being physically rotated anticlockwise, the images were rotated clockwise during processing to counteract this and to simulate the linear offset vector being rotated in 90° steps; then, the images containing the four different linear offset vectors were averaged at each pixel. The averaged ratiometric values at each pixel were then plotted as false-color 2D heat maps.

## Results and Discussion

### NP-Functionalized Silica Powder as a SESORS Imaging Template

Powder-based SERS-active imaging templates were designed to allow for a small, precise inclusion point that was optically active in the near-infrared (NIR) biological window for through tissue SESORS imaging experiments. Previous SESORS studies utilized NP-spin-coated glass slides with uniform SERS signals across the sample or NP liquids spotted and dried directly on the tissue.^18,21^ By contrast, the templates used here are simple to prepare, provide reliable and strong SERS signals from a wide range of Raman reporter molecules when probed with a NIR laser, are easily customizable in terms of the inclusion size and concentration in imaging experiments, and retain their favorable optical properties under long-term storage and when buried for several hours within the tissue. They also remain in a defined location, whereas spotting solution-based NPs onto the tissue can result in spreading of the spot during drying as well as NP penetration into the tissue above and below. They are prepared by mixing a silica powder with a solution of SERS-active silica-coated NP aggregates, synthesized with the commercially available Raman reporter molecules PPY and BPE, specifically designed to exhibit a large, reproducible, and unique SERS fingerprint distinguishable from tissue Raman and other NP SERS signatures during SORS measurements, Figure 1D.^26^ Prior to mixing the SERS-active nanotags with the silica powder, the colloidal solutions were characterized using DLS, zeta potential analysis, and extinction spectroscopy, Figure S1. The DLS measurements indicated that the silica-coated NP aggregates were larger than 100 nm, and it is worth noting that although they were intended for demonstration of a strong SERS response using NIR excitation wavelengths, NPs of this size would be unsuitable for in vivo applications because they would stimulate an immune response within the human body.^27^ The silica-coated NP aggregates feature a localized surface plasmon resonance at 530 and 535 nm, and low-intensity extinction bands from aggregated NPs were observed at 715 and 770 nm for BPE and PPY, respectively. These extinction bands in the NIR spectral window are not present in extinction spectra of the gold NP cores, and they indicate that the NP aggregates are optically active when probed using 785 nm excitation.

### Ratiometric SESORS Imaging with a Variable Linear Offset Reveals Offset-Induced Image Drag

It has previously been established that ratiometric analysis of the subsurface NPs versus the surface tissue Raman intensities, *I*_NP_/*I*_Tis_, is useful for providing information about the depth of NPs within tissue in SESORS measurements.^21,28^ When the depth of the NPs is varied and the offset is fixed, this analysis technique can be used to calibrate the Raman intensity of the NPs against the depth using log-linear regression, and when the offset is varied and the depth is fixed, it allows for different depths to be probed within the NP/tissue system. Therefore, it would be highly advantageous to apply ratiometric analysis to SESORS imaging to not only allow for determination of the location of a SERS-active inclusion, and hence a disease state, in the *x*, *y*-imaging plane but also gain information about the inclusion location in the *z*-axis, that is, the depth. A comparison between intensity and ratiometric SESORS imaging is provided in Figure S2.

**Figure 2:**
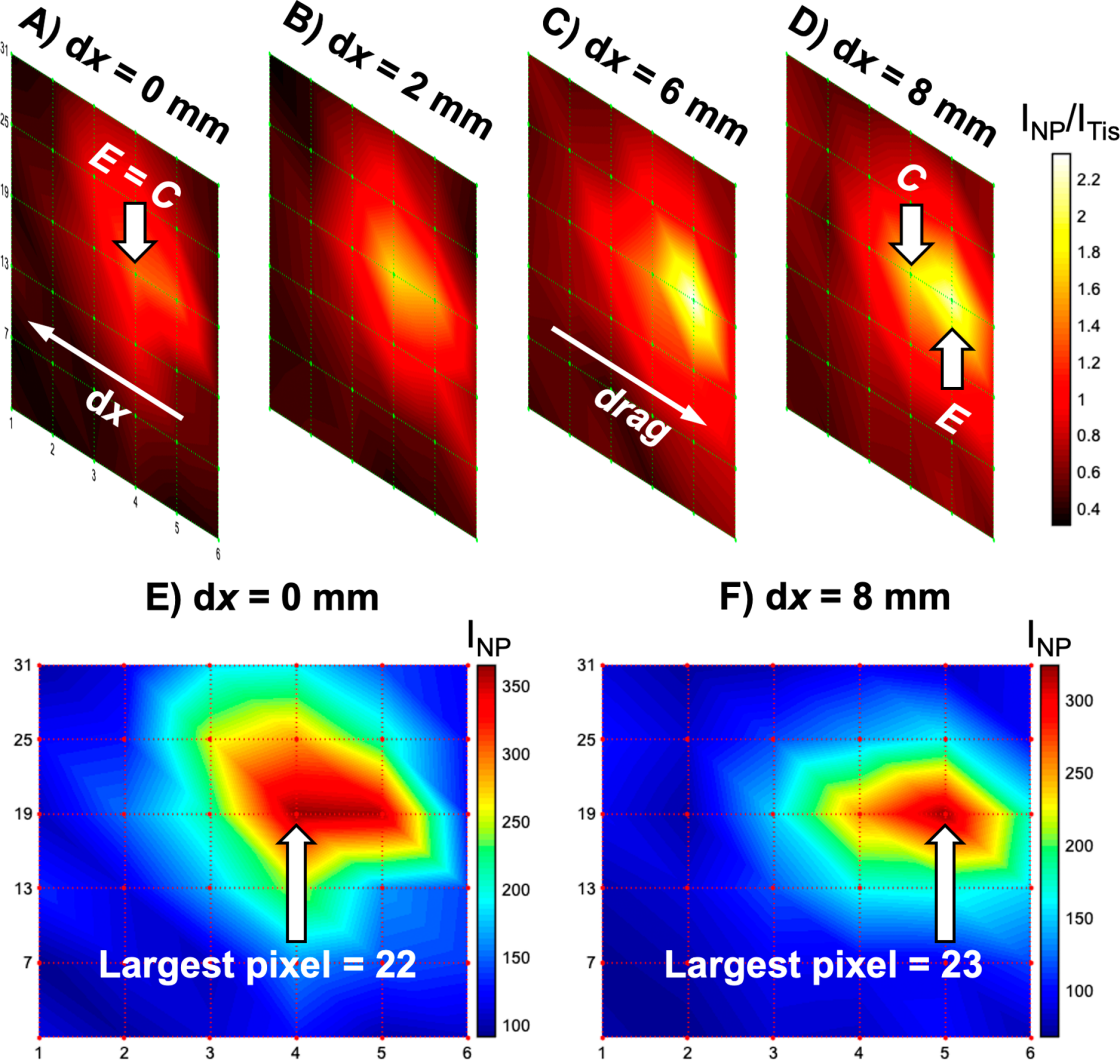
Ratiometric SESORS imaging with a variable offset reveals the effect we termed as linear offset-induced image drag. To observe this, the PPY powder was buried 15 mm behind the tissue barrier, and images were collected independently on an in-house-built fixed SORS system across a 25 × 25 mm grid in 5 mm steps with linear offset magnitudes of (A) 0, (B) 2, (C) 6, and (D) 8 mm. Note that in (D), the collection point is highlighted for the highest intensity pixel, i.e., the excitation point where the largest intensity ratio was detected. The color bar across the set of offset images was scaled for clarity to indicate the offset that maximized the intensity ratio, *I*_NP_/*I*_Tis_. Intensity SESORS images, where each pixel corresponds to I_NP_, were recorded with linear offset magnitudes of (E) 0 and (F) 8 mm to clearly display the effect of linear offset-induced image drag. Spectra were collected for 1 s using a laser power of 400 mW.

The ability of ratiometric SESORS imaging to probe different depths through tissue using NPs at a fixed depth and incrementally varying spatial offsets was investigated using the in-house-built SORS spectrometer. The silica powder mixed with silica-coated PPY-functionalized gold NPs (PPY powder) was buried 15 mm behind the tissue barrier, and ratiometric images were collected across a 25 × 25 mm grid in 5 mm steps with linear offset magnitudes of 0, 2, 6, and 8 mm, Figure 2A–D. The color bars were aligned across all variable offset images from each experiment for clarity. Since the intensity ratio *I*_NP_/*I*_Tis_ can be modeled as a function of the tissue barrier thickness and the spatial offset magnitude, it follows logically that the intensity ratio should be maximized at an offset proportional to the depth at which the NPs are buried behind the tissue barrier. For example, the intensity ratio would be maximized at a small offset magnitude when the NPs are buried close to the surface–air interface and vice versa. This result is in accordance with previous work that noted a strong correlation, the nature of which varies with the scattering properties of the turbid medium, between the SORS-probed depth and the spatial offset applied.^22^ This phenomenon was observed, and the intensity ratio of the high-intensity areas within the images was maximized at the larger spatial offset magnitudes.

It was also observed that as the spatial offset magnitude was increased, the maximum intensity area within the images moved relative to the images collected with no spatial offset. This can be observed more clearly in the corresponding intensity SESORS images recorded at linear offset magnitudes of 0 mm and 8 mm, Figure 2E,F, where the maximum intensity pixel clearly changes between the images collected with different linear offset magnitudes. It was reasoned that the 0 mm offset image, Figure 2A,E, indicated the true location of the powder inclusion in the *x*, *y*-imaging plane since the excitation and collection probes were focused on the same position on the tissue surface at each pixel, and any Raman-scattered photons from the subsurface NPs would only be collected at a pixel if the SERS-active inclusion was present in that location. It is crucial to note throughout this study that the Raman intensity at any given pixel is related to where the sample was excited and not collected because when a spatial offset is applied, these two points become separated. The directionality of this effect was compared to the direction of the linear offset vectors on the set of images, that is, the path from the excitation point, E, to the collection point, C. The direction of the offset vector was calculated by mapping each pixel from the physical experiment onto the final image and mapping the physical position of the excitation and collection probes in the same way. Inclusion of the linear offset vector in the set of ratiometric images revealed the position of the maximum intensity areas within the images. This was noted previously by Botteon et al. in a micro-SORS imaging study of art samples, and the effect can also be observed in variable offset SORS images of a plastic inclusion through bone in the work by Nicolson et al., although in both cases the implication of the effect for the through barrier *x*, *y*-plane localization of subsurface targets was not discussed.^20,29^ We have termed this phenomenon of movement in the image against the offset vector as “linear offset-induced image drag”. This is observed in SESORS imaging when a linear offset is applied because the excitation and collection probes are focused on different areas on the tissue surface, and the photons follow a linear path through the tissue medium between the two spots.^30,31^ Therefore, if a SERS-active NP inclusion were to lie within the axis of the linear offset vector, it would likely be excited, and hence, the resulting Raman-scattered photons would be detected. Linear offset-induced image drag occurs in the direction opposite to the linear offset vector because an inclusion may be detected when it is buried beneath the collection point, that is, it will be detected even when the excitation point is offset from the real location of the inclusion. Linear offset-induced image drag is shown and described further in Figure S3. The discrepancy between the two probes used in SESORS imaging is shown in Figure 2D, where it can be observed that the ratiometric image collected with a linear offset of 8 mm is subject to linear offset-induced image drag because the inclusion is physically offset from where the pixels are recorded, and they are excited below the collection point at these specific pixels. It is hence implied that because of this effect, a SESORS image and indeed a SORS image created using a linear offset vector do not accurately indicate the location of a SERS-active NP inclusion in real physical space, and hence, a backscattering point-collection SORS geometry should be limited primarily to through barrier detection methods where the accurate location in the *x*, *y*-plane is not an objective.

**Figure 3:**
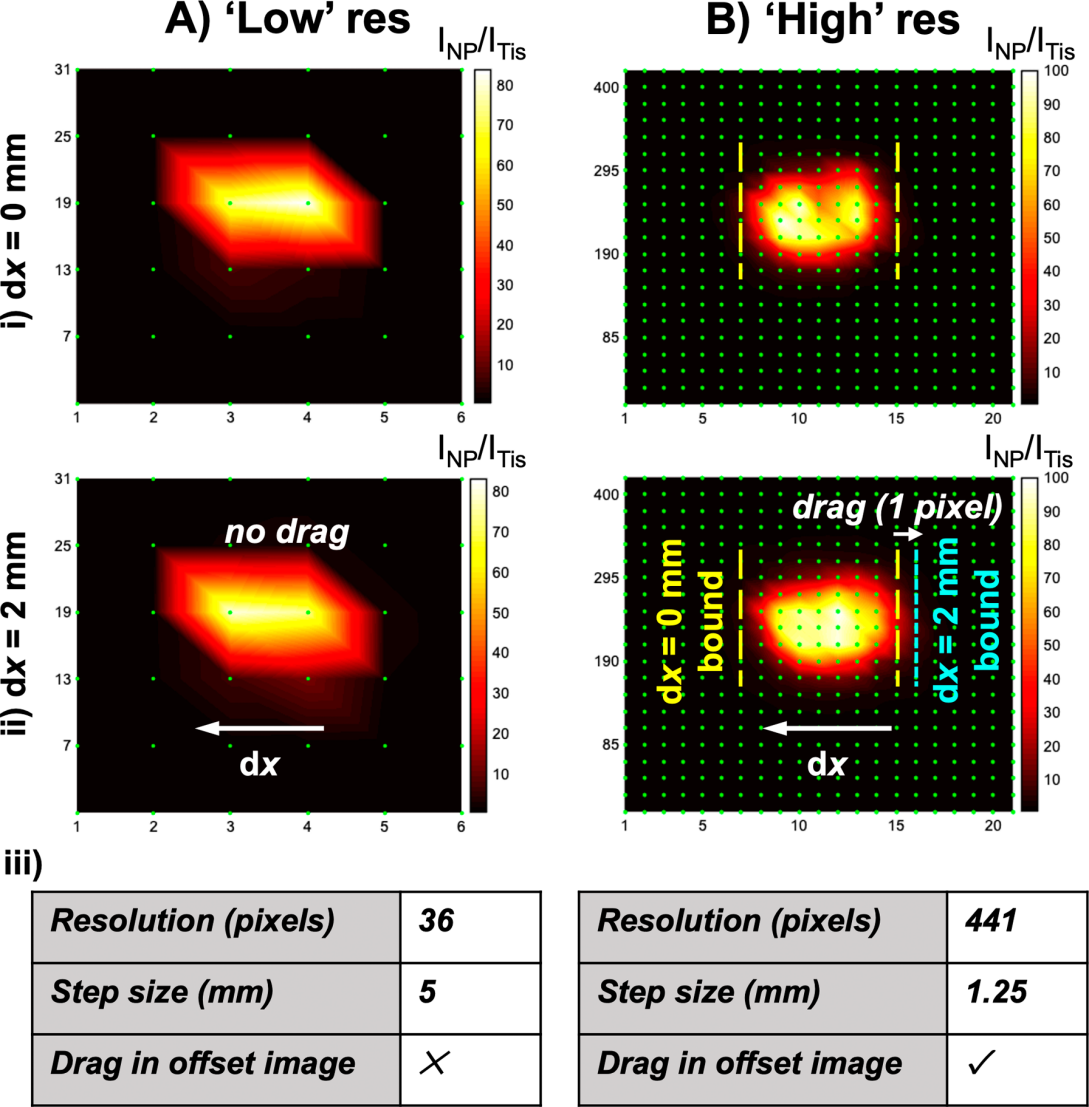
High-resolution ratiometric SESORS imaging reveals proportionality between linear offset-induced image drag and the magnitude of the applied offset. PPY powder was buried behind 3 mm of tissue, and images were collected on the in-house-built SORS system across a 25 × 25 mm grid in (A) 5 mm steps and (B) 1.25 mm steps with linear offset magnitudes of (i) 0 and (ii) 2 mm. (iii) Tables containing information about the resolution of the images and the presence of linear offset-induced image drag in the images collected with an offset magnitude of 2 mm. The color bar across the two sets of offset images was scaled for clarity to indicate the offset that maximized the intensity ratio, *I*_NP_/*I*_Tis_. Spectra were collected for 1 s using a laser power of 400 mW.

High-resolution ratiometric SESORS imaging was performed to ascertain whether the magnitude of the linear offset vector was proportional to the magnitude of linear offset-induced image drag between images. The PPY powder was buried beneath 3 mm of tissue, and images were collected on the in-house-built SORS system across a 25 × 25 mm grid in 1.25 mm steps with linear offset magnitudes of 0 and 2 mm. This was repeated, but the image step size was altered to 5 mm, meaning that the resolution was lower (36 pixels vs 441 pixels). These results can be observed in Figure 3, and they indicate that when the step size used to perform imaging is lower than the offset magnitude used, linear offset-induced image drag occurs in the opposite direction and in proportion to the offset magnitude relative to the image collected with no offset. In the high-resolution images, linear offset-induced image drag can be observed over 1 pixel, corresponding to between 1.25 and 2.5 mm, between the images collected at offset magnitudes of 0 and 2 mm by overlaying and comparing the horizontal boundaries of the high-intensity areas in the two images. Since the step size in the low-resolution images exceeds the magnitude of the linear offset vector used, drag is not observed between the images collected at offset magnitudes of 0 and 2 mm. This means that when SESORS imaging is conducted with a linear spatial offset that exceeds the step sizes taken in the construction of the image, the location of the inclusion from the image is distorted relative to its location in physical space proportionally with the magnitude of the linear offset vector. This is particularly significant when high-resolution images are collected, as would be the case for diagnostic imaging in a clinical setting where the definition of the margin between healthy and diseased tissues is imperative. If not considered, this proportional drag effect would lead to a false depiction of the location of an inclusion, and hence a disease state, at depth.

### Ring-Collection SESORS Imaging as a Means of Removing Linear Offset-Induced Image Drag

**Figure 4:**
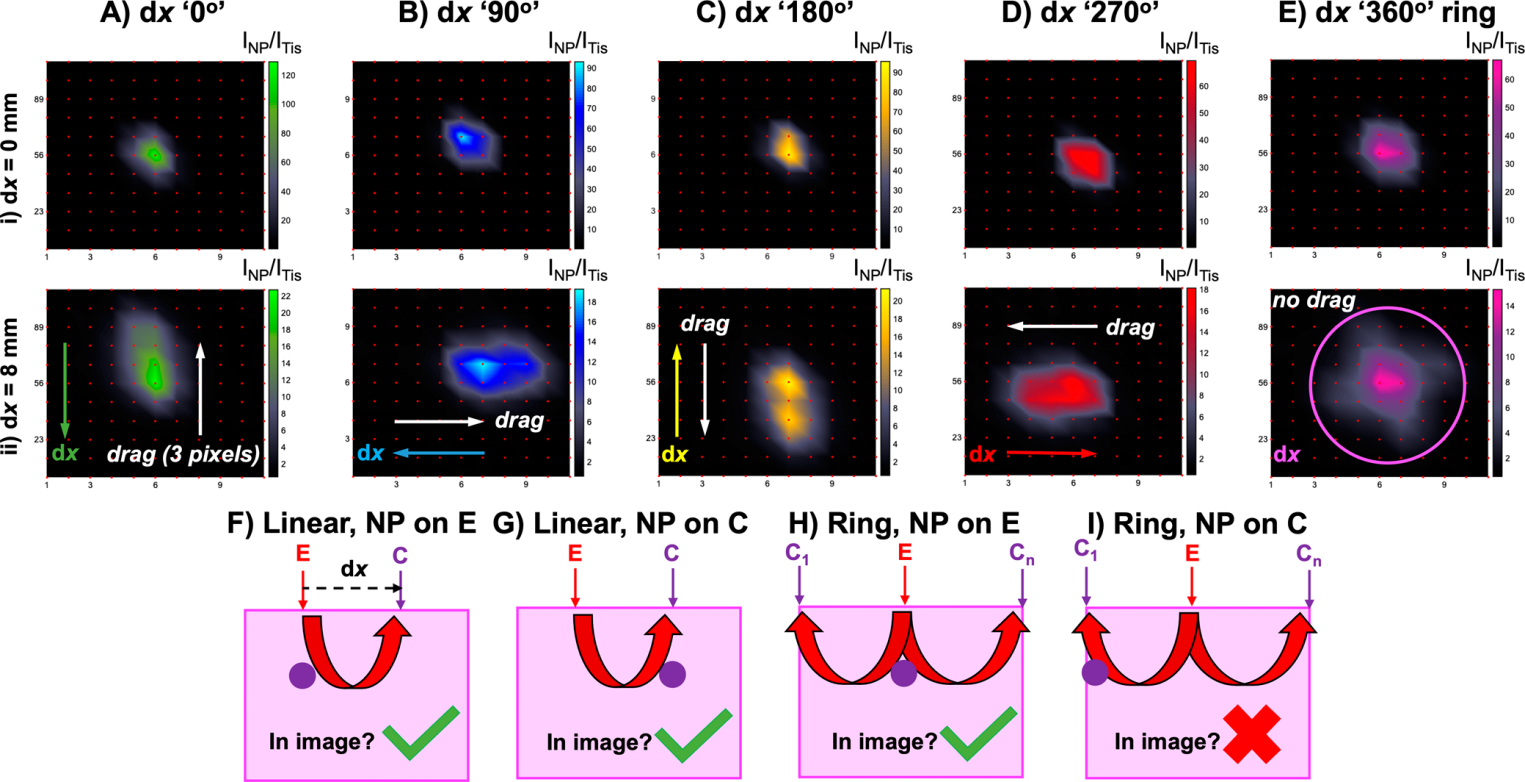
Ring-collection ratiometric SESORS imaging as a means of removing linear offset-induced image drag. (A) Ratiometric images were collected of the PPY powder buried behind 3 mm of tissue across a 25 × 25 mm grid in 2.5 mm step sizes with linear offset magnitudes of (i) 0 and (ii) 8 mm. This was repeated three times with the sample being rotated through 360° in 90° steps to mimic rotation of the linear offset vector, and the resulting vectors were labeled with respect to the orientation of the vector in the initial set of images (d*x* “0°”) as (B) d*x* “90°”, (C) d*x* “180°”, and (D) d*x* “270°”. (E) Ratiometric images averaged across the four linear offset vectors to mimic a ring-collection offset at (i) 0 and (ii) 8 mm. (F–I) Schematics showing the effect of the optical geometry on the appearance of a through tissue SORS/SESORS image. Spectra were collected for 1 s using a laser power of 400 mW.

From the previous sections, it is evident that steps must be taken to eradicate linear offset-induced image drag for the correct location of SERS-active NP inclusions through tissue. Linear offset-induced image drag occurs because of asymmetry in the imaging process, and to remove this effect, it is necessary to either correct for it numerically, which is possible because previous work stipulates that the maximum signal in a linear SORS measurement is typically placed halfway between the excitation and collection points, or by adopting an alternative optical geometry.^29,32^ Initial numerical studies of SORS specified that when using a ring-collection SORS geometry, spectral data could be improved significantly over a point collection SORS geometry in terms of the signal-to-noise ratio.^33^ Additionally, since this optical setup is symmetrical and involves the collection of photons from 360° around a central excitation point, it was hypothesized that it could be adopted to remove asymmetrical linear offset-induced image drag. This section investigates the effect that a mimicked ring-collection spatial offset has on the location of NP inclusions in ratiometric images with a variable offset magnitude. We sought to ascertain if it would be a better indicator of the location of NPs through the tissue in the *x*, *y*-imaging plane by introducing a rotational variable in imaging experiments. A ring-collection SORS geometry was imitated by mounting a rotational platform with a range of 360° beneath the optical setup of the in-house-built SORS spectrometer and on top of the previously used *x*, *y*, *z*-translational stage. Ratiometric images were collected of the PPY powder buried behind 3 mm of tissue across a 25 × 25 mm grid in 2.5 mm step sizes with linear offset magnitudes of 0 and 8 mm. This was repeated three times with the sample being rotated through 360° in 90° steps to mimic the rotation of the linear offset vector. Since the samples were being physically rotated anticlockwise, the images were rotated clockwise during processing to counteract this and to simulate the linear offset vector being rotated in 90° steps, as can be observed in Figure 4. It is evident in this set of images, Figure 4A–4D, that linear offset-induced image drag occurs in the opposite direction but is proportional to the linear offset vector in all instances between the images collected with offset magnitudes of 0 and 8 mm. Then, to imitate a ring-collection SORS image, the images containing the four different linear offset vectors were averaged at each pixel, and the resulting images, Figure 4E, indicate that asymmetric linear offset-induced image drag is removed between the images collected with offset magnitudes of 0 and 8 mm. The location of the inclusion in the two averaged images is identical, suggesting that a ring-collection SORS geometry is more suitable for inclusion location in the *x*, *y*-imaging plane than a linear point-based collection SORS geometry.

To explain why a ring-collection SORS geometry removes linear offset-induced image drag and is a better indicator of the inclusion location through the tissue than point-based collection, a visual aid showing four hypothetical scenarios is provided in Figure 4F–4I. These show imaging schematics with two variables in different permutations, namely, the geometry of the spatial offset and the location of the NP inclusion with respect to the excitation and collection probes. Schematics in Figure 4F,G use a linear point-based collection SORS geometry, where the excitation and collection probes are focused on two points on the tissue surface separated by a linear offset vector. In schematic Figure 4F, the inclusion is located below the spot on which the excitation probe is focused, and in schematic Figure 4G, the inclusion is located below the spot on which the collection probe is focused. The red arrows in all of the schematics are indicative of the path that the laser-generated photons undergo between the spots on which the optical probes are focused, and this was previously reported in studies that employed Monte Carlo simulations to characterize photon migration in turbid media.^22,30,31^ Schematics in Figure 4F,G show that, as previously discussed, due to the propagation of the photons between the two spots, the NPs will appear in an image when buried underneath both the excitation and collection probe-focused spots, and this is the source of linear offset-induced image drag. However, in schematics of Figure 4H,I, which depict a ring-collection offset that logistically consists of multiple collection-focused spots oriented in a circle around a central excitation point, the inclusion only appears in an image when it is buried beneath the spot on the tissue on which the excitation probe is focused. Linear offset-induced image drag is removed because the inclusion is not detected in an image when it is buried beneath one of the collection probe-focused spots. This is because the Raman and SERS signals from the tissue and NP response collected at each pixel are averaged over multiple collection probe-focused spots, and any SERS signal from the inclusion detected in one collection probe will be dampened over multiple probes. This allows for a more accurate representation of the inclusion point, which is hugely important for non-invasive, in vivo detection using SESORS for clinical diagnostic purposes in the future.

### Probing Through Tissue in Three Dimensions Using Ratiometric SESORS Imaging and a Variable Ring-Collection Offset

**Figure 5:**
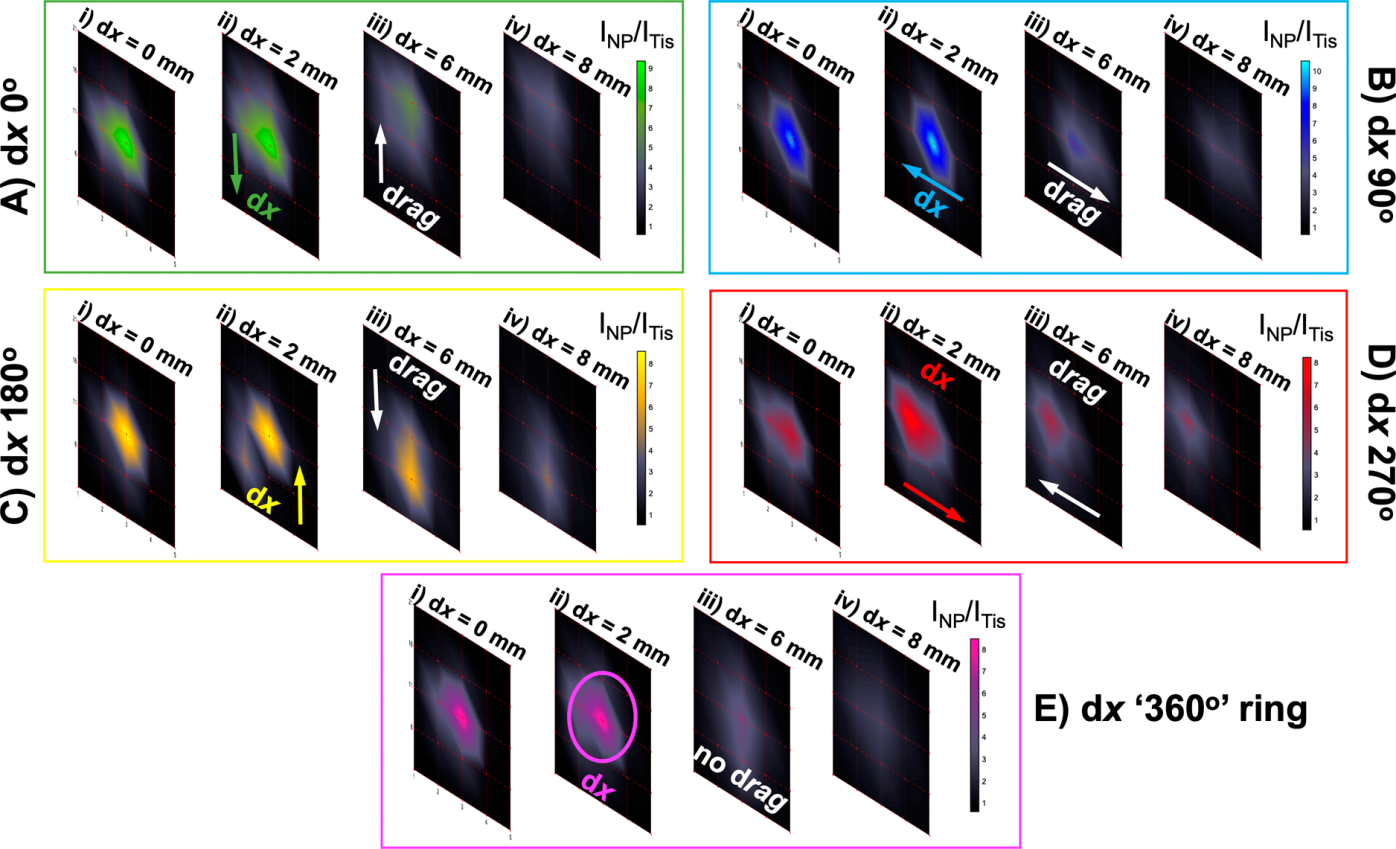
Probing through tissue in three dimensions using ratiometric SESORS imaging and a variable ring-collection offset. (A) Ratiometric images were collected of the PPY powder buried beneath 6 mm of tissue across a 20 × 20 mm grid in 5 mm step sizes with linear offset magnitudes of (i) 0, (ii) 2, (iii) 6, and (iv) 8 mm. This was repeated three times with the sample being rotated through 360° in 90° steps to mimic the rotation of the linear offset vector, and the resulting vectors were termed with respect to the orientation of the vector in the initial set of images (d*x* “0°”) as (B) d*x* “90°”, (C) d*x* “180°”, and (D) d*x* “270°”. (E) Ratiometric images averaged across the four linear offset vectors to mimic a ring-collection offset at (i) 0, (ii) 2, (iii) 6, and (iv) 8 mm. The color bars across each set of offset images were scaled for clarity to indicate the offset that maximized the intensity ratio, *I*_NP_/*I*_Tis_. Spectra were collected for 1 s using a laser power of 400 mW.

It follows from the previous sections that ratiometric SESORS imaging with an increasing ring-collection offset magnitude should allow 3D tracking of NP inclusions through the depth axis in a fixed NP/tissue system. Here, this hypothesis was tested through the ratiometric imaging of the PPY powder buried at a depth of 6 mm beneath the tissue using the in-house-built SORS system across a 20 × 20 mm grid in 5 mm step sizes with mimicked ring-collection offset magnitudes of 0, 2, 6, and 8 mm. Again, to do this, ratiometric images of the powder were collected with the four linear offset magnitudes and a constant linear offset vector direction. This was repeated three times with the sample being rotated through 360° in 90° steps to mimic the rotation of the linear offset vector; then, to imitate ring-collection SORS images, the images containing the four different linear offset vector directions and the same offset magnitude were averaged at each pixel. The results of this are shown in Figure 5. In the ratiometric images that were acquired when a linear offset was applied and incrementally increased, linear offset-induced image drag can be observed. However, when the linear offset directions are averaged out to a ring-collection offset, the NPs can be probed through the tissue without linear offset-induced image drag and are in the same location, and the maximum intensity pixel is the same across all the images as the offset magnitude is varied. The ratiometric images also demonstrate that the contribution of the NPs is maximized in SORS spectra at the smaller offset magnitudes of 0 and 2 mm, indicating that a small offset magnitude is more suitable for focusing on inclusions buried behind small tissue barriers.^21,22^ At the larger offsets used, the contribution of the NPs in the spectra and images is lower, indicating that these offsets are too large, and the excitation photons are focused on depths beneath the NP inclusions. This has the potential to be a new methodology for detecting the location of a NP inclusion in the 2D *x*, *y*-imaging plane using the ring-collection offset to remove image drag and in the *z*-depth plane by altering the magnitude of the ring-collection offset. Unlike an alternative method developed by the authors that requires internal calibration of the NP SERS intensity, this technique would not require prior knowledge of the NP location because it is primarily affected by the parameters of the optical setup.^28^ However, associating a specific depth in a quantitative manner with experimental observables would still require knowledge of the optical properties of the tissue and parallel photon propagation simulations because the relationship between the spatial offset magnitude and the penetration depth of the laser has been previously found to be dependent on the scattering properties of the sample matrix.^22^

### 3D Imaging and Spatial Discrimination of Two Flavors of SERS NPs Simultaneously Using Ring-Collection Ratiometric SESORS

**Figure 6:**
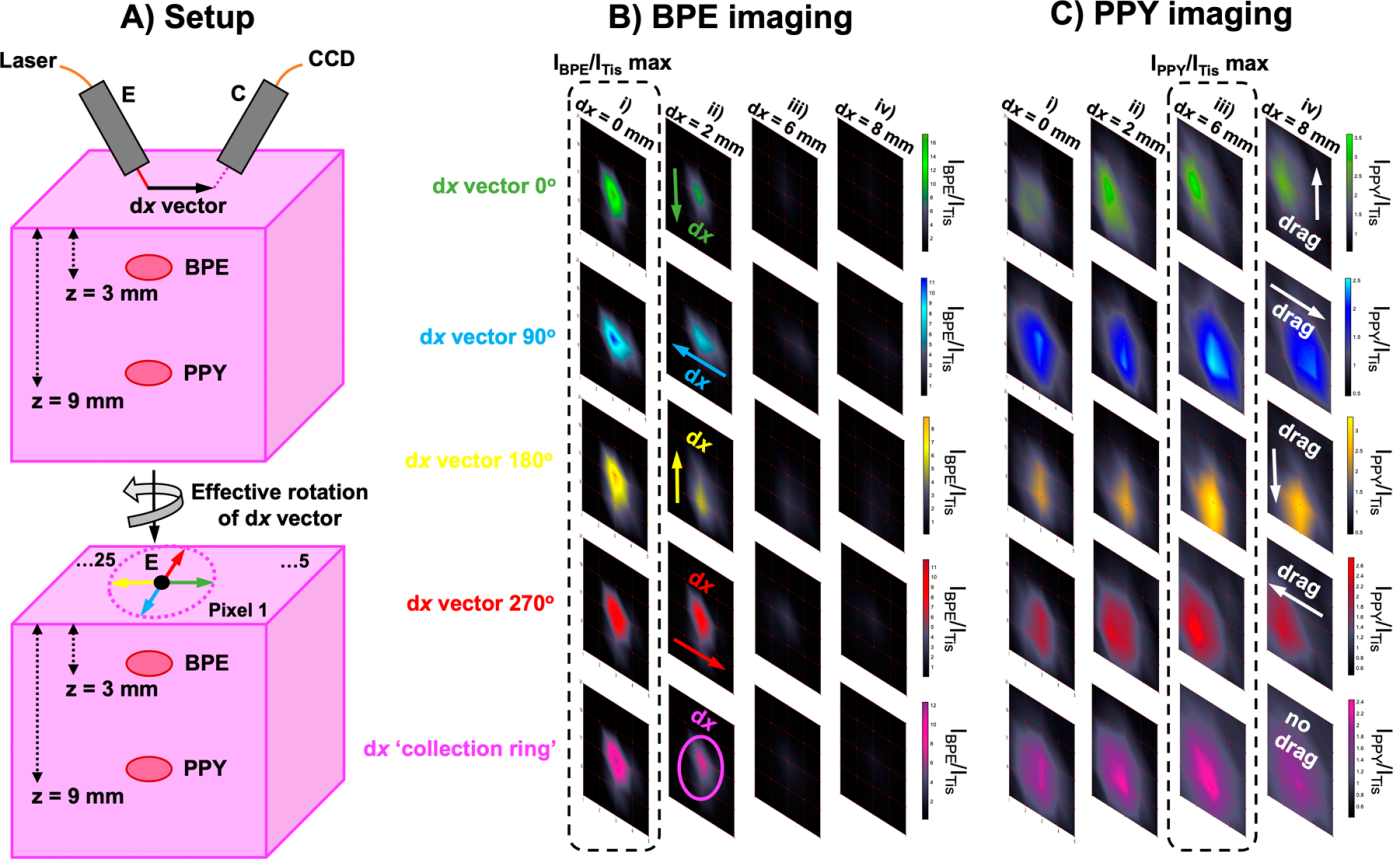
3D imaging and spatial discrimination of two flavors of SERS nanotags simultaneously using ring-collection ratiometric SESORS. (A) Schematic showing the spatial discrimination experiment setup; images were collected of the BPE and PPY powders beneath 3 and 9 mm of tissue in the same sample across a 20 × 20 mm grid in 5 mm step sizes with various linear offset magnitudes. This was repeated three times with the sample being rotated through 360° in 90° steps to mimic the rotation of the linear offset vector, and the resulting vectors were termed with respect to the orientation of the vector in the initial set of d*x* “0°” (green) images as d*x* “90°” (blue), d*x* “180°” (yellow), and d*x* “270°” (red). Ratiometric images were averaged across the four linear offset vectors to mimic a ring-collection offset (pink). (B) Ratiometric images were collected of the BPE powder buried beneath 3 mm of the tissue across a 20 × 20 mm grid in 5 mm step sizes with linear offset magnitudes of (i) 0, (ii) 2, (iii) 6, and (iv) 8 mm. This was repeated three times with the sample being rotated through 360° in 90° steps to mimic the rotation of the linear offset vector, and the resulting vectors were termed with respect to the orientation of the vector in the initial set of d*x* “0°” (green) images as d*x* “90°” (blue), d*x* “180°” (yellow), and d*x* “270°” (red). The ratiometric images were averaged across the four linear offset vectors to mimic a ring-collection offset (pink) at each offset magnitude. (C) Ratiometric images were collected of the PPY powder buried beneath 9 mm of the tissue across a 20 × 20 mm grid in 5 mm step sizes with linear offset magnitudes of (i) 0, (ii) 2, (iii) 6, and (iv) 8 mm. This was repeated three times with the sample being rotated through 360° in 90° steps to mimic the rotation of the linear offset vector, and the resulting vectors were termed with respect to the orientation of the vector in the initial set of d*x* “0°” (green) images as d*x* “90°” (blue), d*x* “180°” (yellow), and d*x* “270°” (red). The ratiometric images were averaged across the four linear offset vectors to mimic a ring-collection offset (pink) at each offset magnitude. The color bars across each set of ratiometric images were scaled for clarity to indicate the offset that maximized the intensity ratio, *I*_NP_/*I*_Tis_. Spectra were collected for 1 s using a laser power of 400 mW.

Ratiometric SESORS imaging with an increasing ring-collection offset magnitude has been presented for 3D tracking of NP inclusions through a depth axis in a fixed NP/tissue system. To test the robustness of this method for 3D ratiometric imaging and inclusion location, we sought to achieve spatial discrimination between two “flavors” of SERS NPs, that is, the silica powder mixed separately with different Raman reporters, placed at different depths, *z*-plane, but at an equivalent position in the *x*, *y*-imaging plane, within a single tissue model. Since the spatial offset magnitude can be related to the depth probed by the laser, it follows that ratiometric analysis of two different NP inclusions buried at different depths should yield maximum NP contributions at separate offset magnitudes, thereby separating the two inclusions in three dimensions. To achieve this spatial discrimination, samples consisting of the silica powder mixed with silica-coated BPE-functionalized gold NPs (BPE powder) and the PPY powder were buried separately beneath 3 mm (BPE) and 9 mm (PPY) of tissue and imaged using the in-house-built SORS system across a 20 × 20 mm grid in 5 mm step sizes with mimicked ring-collection offset magnitudes of 0, 2, 6, and 8 mm. Care was taken to ensure that the PPY and BPE powders were placed at the same position in the *x*, *y*-imaging plane, so they were only separated in the depth axis. Ratiometric images of the two flavors were collected with the four linear offset magnitudes and a constant linear offset vector direction. This was repeated three times with the sample being rotated through 360° in 90° steps to mimic the rotation of the linear offset vector. For ring-collection SESORS images, the images containing the four different linear offset vector directions for the same offset magnitude were averaged at each pixel. The results of this section are shown in Figure 6.

Since the two flavors of nanotags were buried at different depths within the single tissue model, the combination of techniques allowed discrimination between the two samples in a 3D space using ratiometric analysis and the mimicked ring-collection SORS geometry. The resulting ratiometric images for the BPE powder show a maximum contribution of SERS NPs at the 0 mm offset, in line with previous experiments in this study, since the BPE powder was placed at a small tissue depth of 3 mm.^34^ The maximum contribution from the PPY powder occurred at the larger 6 mm offset since it was placed at a depth of 9 mm. These results and indeed all of the results herein that utilize ratiometric analysis for depth determination are broadly comparable to the theoretical findings and experimental studies on plastic models in the study by Mosca et al., in which the median, 10, and 90% quantiles of SORS-probed depths are determined as a function of linear spatial offset magnitude in samples with different scattering properties. Deviations from these models can be explained by the more limited number of spatial offset magnitudes investigated, the different optical arrangement used, and heterogeneity in the optical properties of the ex vivo porcine tissue.^22^ It is likely that with a larger number of measurements, this ratiometric analysis technique would demonstrate a maximum contribution of BPE at a spatial offset magnitude between 0 and 2 mm. However, it is clear that these two observations together suggest spatial discrimination of the two samples in three dimensions. This result reinforces the potential that SESORS imaging holds for multiplexed in vivo detection because the experiments conducted not only detect and classify multiple vibrational fingerprints at depth but also provide a means to locate multiple targets simultaneously in three dimensions.^19^ This has implications for a wide range of clinical applications, including cancer imaging, infection diagnostics, and drug delivery monitoring.^9,35−38^

## Conclusions

In summary, we have conducted a study examining experimental techniques for the correct interpretation of SESORS images and outlined a methodology for the accurate location of targeted NPs at depth in three dimensions, which could be applied to the detection of any relevant disease state. The work investigated multiple factors pertaining to the optical settings within through tissue imaging experiments on an in-house-built point-collection-based SORS system and reported the effects of the spatial offset magnitude and geometry in locating SERS-active NPs through the tissue. We presented the effect of linear offset-induced image drag which refers to a spatial distortion in SESORS images caused by the magnitude and direction of the linear offset vector and then utilized a ring-collection-based SORS geometry to remove asymmetric drag effects in images to locate a NP inclusion more accurately in two dimensions. Additionally, to allow for the location of an inclusion through tissue in three dimensions, we introduced the concept of ratiometric SESORS imaging which utilizes the widely reported relationship between the magnitude of the applied offset and the depth probed by the laser. This relationship can be observed experimentally by conducting ratiometric analysis of the subsurface NP and tissue barrier Raman intensities (*I*_NP_/*I*_Tis_), and it was this ratiometric intensity value that was used for the quantification of pixels in SESORS imaging experiments. Combining the principles of ratiometric analysis and linear offset-induced image drag minimization, we ultimately imaged and spatially discriminated between two distinct NP flavors buried at different depths in three dimensions for the first time. This is a significant step forward in the field of SESORS and through tissue detection, and to the best of our knowledge, it is the first time that theoretical principles have been defined for the universal interpretation of SESORS images, which have the potential to be applied to in vivo research and diagnostics in a clinical setting in the future.

## Supporting Information Available

Materials characterization using DLS, zeta potential analysis, and extinction spectroscopy, ratiometric imaging validation, and linear offset-induced image drag demonstration (PDF)

## Notes

Research data associated with this work will become available through the following link: https://doi.org/10.15129/8832bdf0-eeb6-4c13-808c-56bacd59b83a.
