# Supplementary material for: Tomographic Imaging and Localization of Nanoparticles in Tissue Using Surface-Enhanced Spatially Offset Raman Spectroscopy
Source: ACS Appl Mater Interfaces. 2022 Jul 8;14(28):31613–24. doi: 10.1021/acsami.2c05611 (PMC9305698; doi:10.1021/acsami.2c05611)
Supplement: Supplementary file 1 — am2c05611_si_001.pdf [file am2c05611_si_001.pdf]

Supporting Information

Tomographic Imaging and Localisation of Nanoparticles in Tissue Using Surface Enhanced Spatially Offset Raman Spectroscopy

Matthew E. Berry, [†, a] Samantha M. McCabe, [†, a] Sian Sloan-Dennison, [a] Stacey Laing, [a] Neil C. Shand, [b] Duncan Graham [a] and Karen Faulds [a]*

[a.] *Department of Pure and Applied Chemistry, Technology and Innovation Centre, University of Strathclyde, 99 George Street, Glasgow, G1 1RD, UK.*

[b.] *The Defence Science and Technology Laboratory (Dstl), Porton Down, Salisbury, SP4 0JQ, UK.*

SESORS Imaging Template Precursor Analysis

A)

Size	Reading 1	Reading 2	Reading 3	Average ± Polydispersity Index
Au-BPE@SiO_2	111.7	113.7	111.8	112.4 ± 1.13
Au-PPY@SiO_2	105.5	116.3	106.5	109.4 ± 0.44

B)

Zeta Pot	Reading 1	Reading 2	Reading 3	Average ± Standard Deviation
Au-BPE@SiO_2	-42.5	-44.6	-44.8	-44.0 ± 1.27
Au-PPY@SiO_2	-33.8	-38.0	-36.6	-36.1 ± 2.14

C)

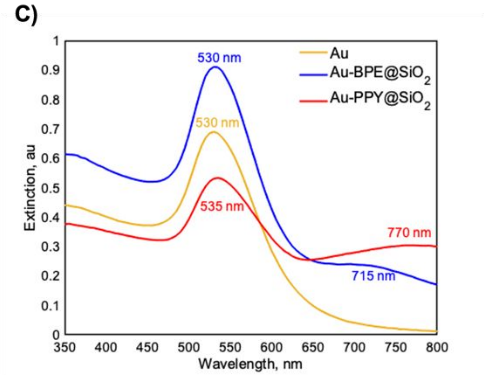

Figure S1. A) Dynamic light scattering (DLS) and B) zeta potential analyses C) Extinction spectra of bare gold NPs prior to reporter addition and silica encapsulation (yellow; 400 µL; dilution factor 2) and silica coated SERS active NPs functionalized separately with BPE (blue; 400 µL; dilution factor 4) and PPY (red; 400 µL; dilution factor 5).

Validation of Ratiometric SESORS Imaging Versus Intensity Imaging

To provide a comparison between intensity and ratiometric SESORS imaging, silica powder mixed with silica coated PPY functionalized gold NPs (PPY powder) was buried beneath 9 mm of tissue and images were collected on the in-house built SORS system across a 20 mm x 20 mm grid in 5 mm steps with a linear offset magnitude of 4 mm, Figure S2.

There are two valid methods of ratiometric analysis in the images presented in Figure S2, termed here as the linear equation solution and the scalar solution based on the data processing techniques applied and shown in B) and C) respectively. The main difference between the two methods is that the linear equation solution calculates intensity ratios by dividing I_{NP} from every pixel with only the largest value for I_{Tis} across the image, and the scalar solution calculates the intensity ratios by dividing I_{NP} at each pixel with the value of I_{Tis} at the same pixel and therefore images processed in this fashion are influenced by variations in I_{Tis} across the imaging grid. It can be observed that the linear equation solution provides an image that is identical to the intensity image in terms of features, because it is unaffected by small variations in the tissue signal across the imaging grid. Therefore, not only can ratiometric SESORS images processed using the linear equation solution provide equivalent information to intensity images regarding *x*, *y*-imaging plane location, but importantly they provide an additional means to relate images to the buried depth of the NPs and to probe the fixed system by altering the magnitude of the spatial

offset, bringing SESORS imaging closer to inclusion location in 3-dimensions. It is for this reason that linear equation ratiometric images were deemed to be the most appropriate and all ratiometric images depicted in this study were processed using this method.

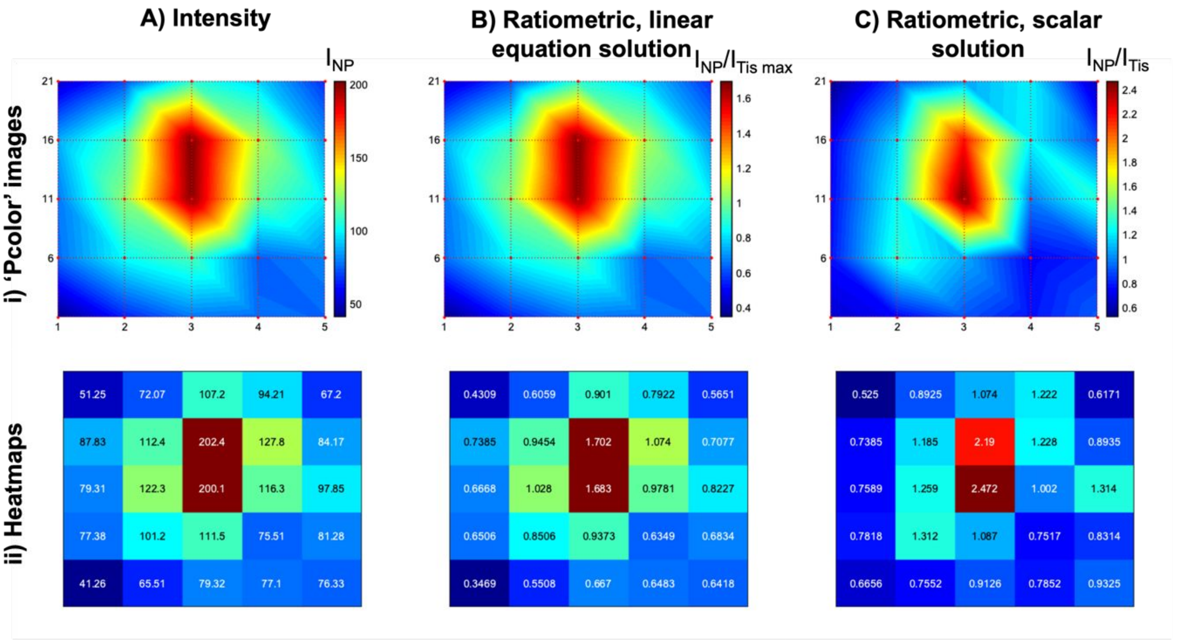

Figure S2. A comparison between SESORS imaging of the NP intensity, I_{NP}, and ratiometric analysis of I_{NP} versus the intensity of the tissue barrier, I_{Tis}. A) Intensity SESORS images (I_{NP}) represented via i) a pseudo color image and ii) a heatmap. B) Linear equation solution ratiometric SESORS images ($I_{NP}/I_{Tis\ max}$) represented via i) a pseudo color image and ii) a heatmap. C) Scalar solution ratiometric SESORS images (I_{NP}/I_{Tis}) represented via i) a pseudo color image and ii) a heatmap. Imaging was conducted by burying PPY powder beneath 9 mm of tissue and collecting spectra on the in-house built SORS system across a 20 mm x 20 mm grid in 5 mm steps with a linear offset magnitude of 4 mm. Spectra were collected for 1 second using a laser power of 400 mW.

<u>Linear Offset Induced Image Drag Schematic</u>

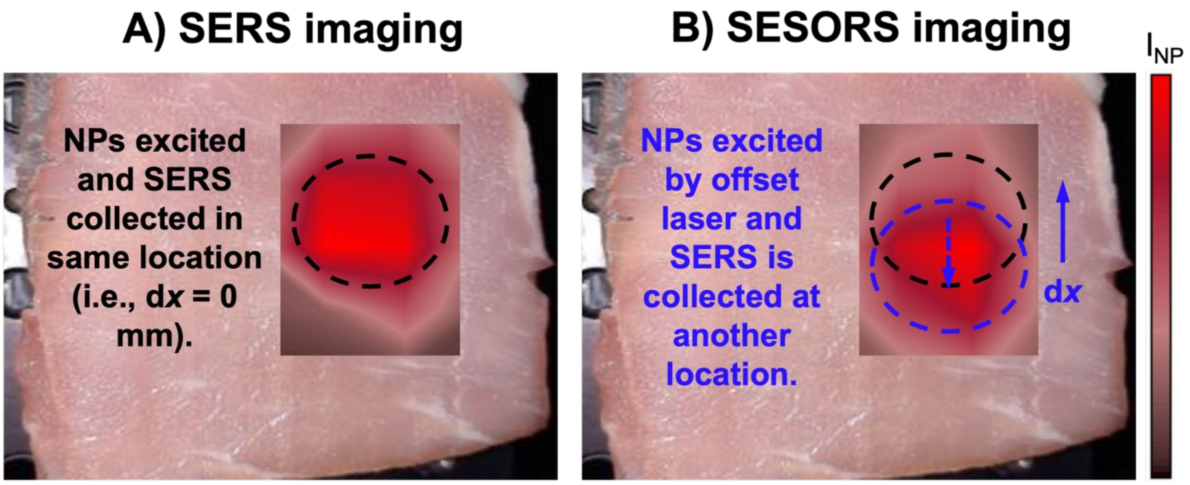

Figure S3. Schematic comparing through tissue SERS and SESORS imaging and portraying linear offset induced image drag. A) Inclusion location from a through tissue SERS imaging experiment, where the excitation and collection probes are focused on the same point on the tissue surface. This implies that any SERS signal from the subsurface NPs originates from NPs excited in the same location, i.e., the location of a NP inclusion in a through tissue SERS image is indicative of the physical location of the inclusion. B) Inclusion location from a point-based

collection through tissue SESORS imaging experiment, where the excitation and collection probes are focused on two spots on the tissue surface separated by a linear offset vector. It can be observed that the location of the NP inclusion in this image is offset from the inclusion location in the through tissue SERS image. It has previously been reported that photons follow a linear path through the tissue medium between the two spots on which the excitation and collection points are focused.[1–3] If a SERS active NP inclusion were to lie within this path, that is on the axis of the linear offset vector, it would be excited by the propagating laser photons and hence the resulting Raman scattered photons would be detected at the collection point. Linear offset induced image drag occurs in the direction opposite to the linear offset vector in SESORS imaging because SERS signatures from an inclusion are detected when the inclusion is buried beneath the collection point or at any point along the linear offset vector axis/the path of laser photons propagating from the excitation point to the collection point through the tissue , i.e., SERS will be detected even when the excitation point, and hence the pixel in an image, is offset from the real location of the inclusion.

References

1. Mosca, S.; Dey, P.; Salimi, M.; Gardner, B.; Palombo, F.; Stone, N.; Matousek, P. Estimating the Reduced Scattering Coefficient of Turbid Media Using Spatially Offset Raman Spectroscopy. *Anal. Chem.* **2021**, *93*, 3386–3392.
2. Mosca, S.; Dey, P.; Salimi, M.; Gardner, B.; Palombo, F.; Stone, N.; Matousek, P. Spatially Offset Raman Spectroscopy – How Deep? *Anal. Chem.* **2021**, *93*, 6755–6762.
3. Mosca, S.; Dey, P.; Salimi, M.; Gardner, B.; Palombo, F.; Stone, N.; Matousek, P. Non-Invasive Depth Determination of Inclusion in Biological Tissues Using Spatially Offset Raman Spectroscopy With External Calibration. *Analyst* **2020**, *145*, 7623–7629.
